# The influence of interpersonal interaction on consumers’ purchase intention under e-commerce live broadcasting mode: The moderating role of presence

**DOI:** 10.3389/fpsyg.2023.1097768

**Published:** 2023-02-16

**Authors:** Xiaoli Ma, Junna Jin, Yunrun Liu

**Affiliations:** ^1^Department of International Trade, Graduate School of Konkuk University, Seoul, Republic of Korea; ^2^Ocean College, Tangshan Normal University, He Bei, China; ^3^Beijing Normal University-Hong Kong Baptist University United International College (UIC), Zhuhai, China

**Keywords:** interpersonal interaction, perceived value, purchase intention, moderating effect, consumer psychological and technological preferences, consumer

## Abstract

The purpose of this study is to examine the links between interpersonal interaction perception, perceived value and purchase intention in e-commerce live broadcasting in China. The mediating effect of perceived value on the relationship between consumer-anchor interaction (CAI) and consumer-consumer interaction (CCI), and purchase intention is explored. Additionally, the moderating effect of presence on the relationship between perceived value and interpersonal interaction perception is also investigated into. The Hayes’ Process macro is utilized as an analysis tool, and the data are gathered *via* an online survey. It is found that both CAI and CCI are both important in increasing perceived value and purchase intention. Besides, perceived value enhances purchase intention while presence acts as a moderator in the relationship between consumer perceived value and interpersonal interaction perception, making the relationship stronger when presence is high and weaker when presence is low. In this way, the results of the study contribute to the current literature of interpersonal interaction under the mode of e-commerce live broadcasting. Employing interpersonal interaction techniques to improve consumers’ perceived value and purchase intention will also be advantageous to enterprises engaged in e-commerce live broadcasting.

## 1. Introduction

The issue of interpersonal interaction has been receiving a great deal of attention in many academic fields and from the business media during recent years (Ying-Jie and Yang, 2014; Guerreiro, 2020; Chen et al., 2022). The reason is that interpersonal interaction is regarded as a method of building an consumer relationship and enhancing the corporate image as well as providing advantages for enterprise marketing (Wei and Yuan, 2019). Under the mode of e-commerce live broadcasting, enterprises have gradually recognized the significance of interpersonal interaction, and are attempting to better meet consumers’ personalized needs and to offer consumers with personalized services (Nugroho, 2019). Under such a mode, interpersonal interaction tends to be considered along with presence to help enhance consumer value and realize value exchange. Moreover, interpersonal interaction is beneficial for building positive images of products and services of enterprises in the eyes of consumers. It also helps strengthen consumers’ brand awareness, cultivating and establishing a stable loyal consumer base for enterprises (Zhang et al., 2017; Chen et al., 2021; Liu et al., 2021; Bai et al., 2022). When enterprises deal with the intense rivalry brought on by new entrants in the e-commerce live broadcasting industry (Wu and Thao, 2019), interpersonal interaction will also be utilized. Besides, this approach can also benefit the enterprises participating in e-commerce live broadcasting, such as a positive brand image, consumer loyalty and the increase of revisits and recommendations (Ko and Chen, 2020). Thus, interpersonal interaction is significant for the enterprise marketing as well as the consumer behavior (Wang M. et al., 2021; Ye and Liu, 2021).

Due to the competitiveness of the e-commerce live broadcasting industry, consumer loyalty is critical (Hou, 2021). Anchors interact directly with consumers, which influences consumer loyalty (Alharbi and Alhider, 2018). Therefore, under the mode of e-commerce live broadcasting, the communication between enterprises and consumers is turned into the communication between consumers, enabling the word-of-mouth communication among consumers and the endless proliferation of consumers (Cao, 2002). Due to its benefits, the interpersonal interaction strategy has been prioritized by many enterprises in the hopes of preserving their competitive advantages (Ding et al., 2020). Due to its advantages, the approach of interpersonal interaction has been emphasized by many enterprises in the hope of maintaining their competitive advantages (Wang Y. et al., 2021). Contrary to the substantial quantity of consumer-anchor interaction (CAI) research, consumer-consumer interaction (CCI) research has gotten less attention. It is critical to comprehend how consumers’ perception of interpersonal interaction can impact their attitudes because of the significant role that interpersonal interaction plays in the e-commerce live broadcasting industry (Lv, 2020).

Despite growing concerns over interpersonal interaction and the urgent need to analyze consumer perception of interpersonal interaction, few studies (Zhao, 2018) have examined the connection between interpersonal interaction and consumers’ attitudes and behaviors. Even less research has been done on the relationship between consumers’ perception of interpersonal interaction and purchase intention, as well as the underlying mechanisms influencing purchase intention. Our research is designed to investigate the relationship between consumers’ perception of interpersonal interaction and purchase intention, and the role of perceived value as a mediator between them. The response of consumers to the interpersonal interaction in a live broadcasting and their purchase intention may vary depending on factors of perceived value (Liu et al., 2021). In some studies on consumer behavior, presence was used as a factor influencing consumers’ attitudes and behavior. Presence refers to the psychological perception of others in communication. That is, presence is a mental perception produced in a setting of e-commerce live broadcasting (Yang et al., 2021). Hence, consumers’ attitudes and behavior could depend on presence.

To further explore consumers’ interpersonal interaction perception, our research on the China e-commerce live broadcasting industry focuses on the relationships among interpersonal interaction perception, perceived value and purchase intention. With S-O-R model and flow theory as a theoretical background, this study investigates into (1) the relationship between CAI and CCI and perceived value, (2) the relationship between perceived value and purchase intention, (3) the mediating effect of perceived value on interpersonal interaction perception and purchase intention, and (4) the moderating effect of presence on the relationship between interpersonal interaction perception and perceived value. In this way, this study fills in the gap in the existing interpersonal interaction literature by figuring out the underlying influence of interpersonal interaction perception on their purchase intention in China e-commerce live broadcasting.

Based on S-O-R model and flow theory, the study contributes to deepening our understanding of the current literature of interaction by investigating into the influence of interpersonal interaction perception on perceived value and purchase intention. Moreover, by exploring how this phenomenon depends on presence, this study can help enterprises better apply marketing strategies and therefore strengthen their competitiveness. Thus, the investigation has a practical significance for the highly competitive China e-commerce live broadcasting industry.

## 2. Literature review

S-O-R model is also known as Stimulus-Organism-Response theory. The foundational model of S-O-R model is S-R model, which postulates that consumers will experience a series of outcomes as a result of external environment stimulation. S-O-R model was then created with the addition of the Organism (O) as research has progressed. It explains how psychological changes lead consumers to behave convergent or avoidant under the influence of the external environment (Jacoby, 2002). Besides, consumers’ cognition and emotions will also change in response to external environmental stimulation, driving them to engage in activities such as using or purchasing (Tian and Youngsook, 2021). Three variables make up the S-O-R model: S-External Stimulation (involving macroscopic external stimulation and microcosmic external stimulation), O-Organism (involving cognition and emotion variable), and R-Response (consumers’ behaviors generated with internal changes under external stimulus).

Flow theory, derived from psychology, explains the unique psychological state that develops when people are entirely absorbed in one activity. When a person is in a psychological flow state, he is entirely engaged in the current event and instantly weeds out the irrelevant information (Chen and Lin, 2018). In order to increase consumers’ flow experiences, anchors tend to entice consumers to participate in the live broadcast through humorous language, drawing lotteries, coupons, famous stars, real-time bullet screens, etc. The series of strategies encourage consumers to understand the usefulness of the products and trust the anchors, thus promoting sales. Meanwhile, immersed in such a atmosphere, consumers will be more likely to watch the live broadcasting, which will raise their purchase intention.

### 2.1. The relationship between interpersonal interaction and consumers’ perceived value

The essence of interaction is a type of communication that can reflect on and provide feedback on the information of a previous communication (Newhagen and Rafaeli, 1996). Interaction between consumers and anchors as well as that between consumers is referred to interpersonal interaction in e-commerce, which has a significant impact on their behaviors and can accurately predict their attitudes and use intention (Van Noort et al., 2012).

Previous researches have analyzed customers’ attitudes and behaviors so as to predict interpersonal interaction perception (Duan et al., 2015; Tang and Yingkang, 2016; Muralidhar et al., 2017). In order to characterize CAI and CCI, other researchers accepted and adapted the definition of interpersonal interaction from (Man et al., 2021). CAI is defined as the interchange and communication process between anchors and customers about the information and emotional experiences of products under the mode of e-commerce live broadcasting. In a similar vein, CCI refers to an interpersonal interaction related to a comparable process between consumers.

Perceived value connects product value and perception of consumers’ psychology, referring to direct reactions of the consumers to product value and primary motivations to choose and purchase products (Jiang et al., 2016). Especially for products with limited value visibility, it is crucial for consumers to detect the differences between their contents among various competitors. Service is a classic example. In this regard, it is considered as one of the most popular promotion strategies in live broadcasting shopping in e-commerce industry. As is categorized by Sweeney and Soutar, perceived value is divided into three categories: functional value, hedonic value, and social value (Soutar, 2001). Functional value refers to consumers’ perception of a product, its functional attributes, etc. Meanwhile, hedonic value is the emotional and psychological satisfaction that a product lives up to consumers’ expectation while social value refers to the group status or psychological benefits that products provide to consumers. These perception dimensions may have varying influences on consumers’ purchase intention with various products and services.

As for customers’ perceived value, it mostly depends on contexts and can be defined as consumers’ overall evaluation of products and services. It decides how much money consumers are willing to pay for products and services. In the context of e-commerce live broadcasting, perceived value is affected by the degree of information they possess and their relationship with other customers.

### 2.2. The relationship between consumers’ perceived value and purchase intention

Perceived value is a comprehensive evaluation that consumers make after measuring the cost and expected benefits of products and services (Zeithaml, 1988). Scholars proposed two types of values – utilitarian value and hedonistic value. Some of them conducted in-depth analysis of the beneficial effects of perceived value on consumers’ purchase decisions and consumption intention (Pahnila and Warsta, 2010).

In the meanwhile, several scholars such as Petrick and Backman (2002) investigated into the positive effects of perceived value from the perspectives of social value and emotional value on consumers’ social identities and purchase attitudes. Studies from scholars such as xxx have demonstrated that consumers’ perceived value of products and services are influenced by their opinions on the quality. The higher the perceived quality from consumers is, the stronger their willingness to buy is (Parasuraman, 1997). As a result, individuals’ purchase intention can be predicted based on their perceived value (Chen, 2008).

### 2.3. The mediating effect of consumers’ perceived value on the relationship between interpersonal interaction and purchase intention

The functions of perceived value are concluded as follows. In e-commerce live broadcasting, anchors’ timely feedback and the information exchanged between viewers satisfied viewers’ needs for comprehensive information, and increased their satisfaction for live broadcasting.

In addition, consumers’ perceived value can enhance their flow experiences, thus promoting interaction. To enhance flow experience, anchors tend to use humorous and individualized language to attract viewers. Meanwhile, anchors will also show consumers the trial process of products, during which consumers can understand the using effects of products and gain value-added information by learning some useful skills (Yang et al., 2021). These strategies are capable of effectively promoting interpersonal interaction, thereby increasing consumers’ purchase intention by meeting consumers’ needs in multiple dimensions.

### 2.4. The moderating effect of presence on the link between interpersonal interaction and consumers’ perceived value

The term “presence” is used to describe the importance of the attendance and presence of other people during the interaction, and the closeness of people’s relationship (Parker et al., 1976). It alludes to a sense of reality whereby individuals are fully absorbed in the setting produced by a particular medium (Kim and Biocca, 1997). According to psychology, there are two types of presence – physical presence and social presence (Ijsselsteijn et al., 2000). Physical presence describes the feeling of “immersion” induced by certain methods. The term “social presence” mostly refers to the experience of “staying or communicating with people,” the idea of which was later introduced to the research of consumers’ behaviors (Han et al., 2016).

Consumers’ presence can be generated with the following steps. In the shopping live broadcasting, users can see the number of users who are purchasing and commenting in the room in real-time, Besides, users can also enhance their presence by identifying themselves through their nicknames and communication, including comments. As is proved by prior researches of scholars such as Shen and Khalifa (2007), communication facilitates information understanding and improves consumers’ perceived value. Moreover, the voice in the room is also a critical factor for generating the presence, such as background music, anchors’ voice, laughs, etc. (Shen and Khalifa, 2007). It can effectively evoke emotional responses among consumers.

## 3. Methodology

### 3.1. Research model and hypotheses

**Figure 1:**
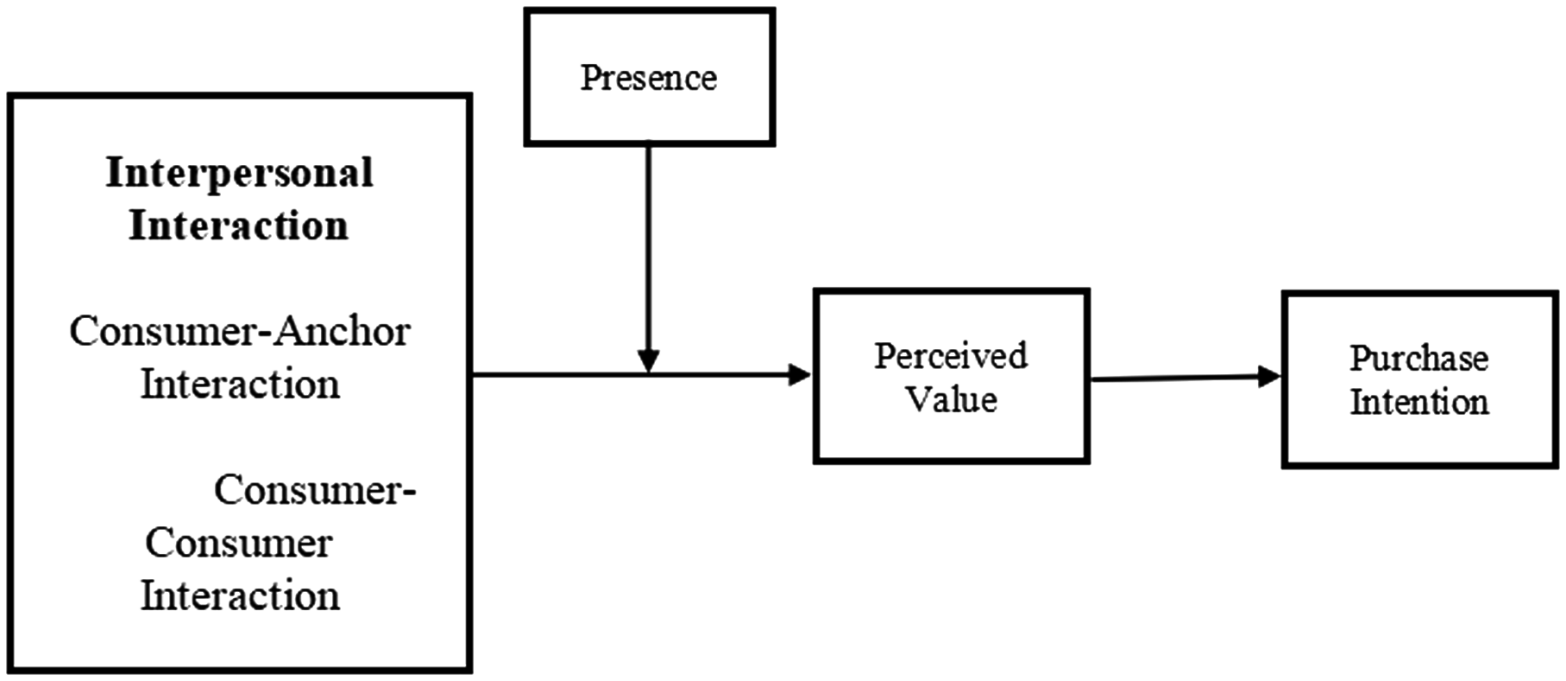
Proposed research model.

Based on S-O-R model and flow theory, the research model (Figure 1) treats perceived value as mediators between interpersonal interaction perception and purchase intention. Additionally, the moderating effect of presence on the relationship between perceived value and interpersonal interaction perception is also explored.

In order to better achieve the research aim, the researchers hypothesize four research questions. The hypotheses of the model are follows:

*Hypothesis 1 (H1a):* CAI relates positively to Consumers’ Perceived Value.

*Hypothesis 1 (H1b):* CCI relates positively to Consumers’ Perceived Value.

*Hypothesis 2 (H2):* Consumers’ Perceived Value relates positively to Purchase Intention.

*Hypothesis 3 (H3a):* Consumers’ Perceived Value mediates the relationship between CAI and Purchase Intention.

*Hypothesis 3 (H3b):* Consumers’ Perceived Value mediates the relationship between CCI and Purchase Intention.

*Hypothesis 4 (H4a):* Presence moderates the relationship between CAI and Consumers’ Perceived Value, such that this relationship will be stronger when the Presence is high and weaker when the Presence is low.

*Hypothesis 4 (H4b):* Presence moderates the relationship between CCI and Consumers’ Perceived Value, such that this relationship will be stronger when the Presence is high and weaker when the Presence is low.

### 3.2. Data and sample

This study used a questionnaire to test the hypothesized model. The questionnaires were posted *via* Sojump,^1^ a large-scale online survey platform in China that is widely used in behavioural and psychological research (Li et al., 2018). We distributed the survey link in a popular livestreaming shopping forum. The respondents were the groups who made livestreaming purchases recently. Participant were asked to recall his or her most recent livestreaming shopping experience before they completed the survey. Finally, 359 valid questionnaires were obtained, with an effective recovery rate of 91.8%. With the survey conducted from 20 July 2022 to 21 September 2022. In basic demographics, 182 participants (50.7%) were male and 177 (49.3%) were female. 130 participants (36.2%) were under the age of 30, with 150 (41.8%) between 31 and 39 years old and 79 participants (22.0%) beyond the age of 40. As for education background, 112 participants (31.2%) were below a bachelor’s degree, and 206 (57.4%) had a bachelor’s degree. The number of participants who had a master’s degree or above are 41 (11.4%). Moreover, 227 participants (63.2%) had 1 to 5 years of experience of online shopping while 80 participants (22.3%) had 6 to 10 years of experience. Only 52 participants (14.5%) had shopped online for more than 10 years.

### 3.3. Measures

Ratings of interpersonal interaction perception, perceived value, purchase intention and presence were based on a five-point Likert scale (1 = not at all likely, 5 = very likely). The three items listed in the section for CAI and four items in the section for CCI were both adapted from the research of Wu and Wu (2006). The four items in the section for perceived value were adapted from the study of Lapierre (2000) while the four items in the presence section were from that of Zhang et al. (2017). Besides, the source of the three items in the section for purchase intention were from the research of Dodds et al. (1991).

The researchers applied confirmatory factor analysis to analyze convergent validity, and Cronbach’s *α* to testify internal consistency so as to evaluate the validity and reliability of the variables on the assessments.

It is showed that Cronbach’s *α* were above 0.7, so all the data were proved reliable (shown in Table 1). The factor loadings of CAI, CCI, perceived value, and purchase intention were all above 0.5 (shown in Table 2).

**Table 1:** Variables and item description.

Variables	Items	Mean (SD)	Factor loading	References
CAI	The anchor can clearly answer my questions and give positive feedback to my opinions in time	3.76 (1.03)	0.80	Ahn et al. (2004) and Wu and Wu (2006)
	The anchor can provide relevant information about my questions		0.78	
	The anchor can communicate and interact with the audience about the product-related information		0.80	
CCI	I can obtain many opinions and suggestions from other consumers’ comments	3.82 (0.99)	0.76	Ahn et al. (2004) and Wu and Wu (2006)
	I can share my shopping experience and feelings with other consumers		0.77	
	Other consumers’ comments can provide me with reference advice		0.76	
	The communication between consumers is adequate		0.73	
Perceived value	I can buy high-quality and inexpensive products in the live broadcasting room	3.91 (0.95)	0.78	Lapierre (2000)
	I can know more details about products		0.77	
	I feel relaxed		0.81	
	I can know some anchors		0.81	
Presence	The information provided in live broadcasting makes me feel like communicating with others	3.77 (0.94)	0.76	Zhang et al. (2017)
	I have the illusion that time passes quickly		0.77	
	The information provided in live broadcasting makes me feel that the products are presented in front of me		0.78	
	The information provided in live broadcasting makes me feel like shopping in a off-line shop		0.74	
Purchase intention	I might buy products in e-commerce live broadcasting	3.76 (1.12)	0.82	Dodds et al. (1991)
	I prefer buying products through e-commerce live broadcasting		0.81	
	I will continue buying products through e-commerce live broadcasting		0.81	

**Table 2:** Correlation and reliability analysis.

	**CAI**	**CCI**	**Perceived value**	**Purchase intention**	**Cronbach’s *α***
CAI	1				0.83
CCI	0.48**	1			0.85
Perceived value	0.33**	0.44**	1		0.85
Purchase intention	0.53**	0.54**	0.42**	1	0.86

*n* = 359, **p < 0.01 (two-tailed test).

### 3.4. Results

The researchers firstly examined whether perceived value mediates the relationship between customers’ interpersonal interaction perception and their purchase intention. The age of the consumers and their prior online shopping experiences were regarded as control variables in previous studies.

To test proposed hypotheses 1, 2 and 3, model 4 from Hayes’ Process macro (Hayes, 2013) was applied in the study. As is predicted, interpersonal interaction perception of CAI related positively to perceived value (*b* = 0.31, SE = 0.04, *p* < 0.01, 95% CI [0.22, 0.40]: H1a supported) while perceived value related positively to purchase intention (*b* = 0.32, SE = 0.05, *p* < 0.01, 95% CI [0.21, 0.43]: H2 supported). In addition, the study indicated that interpersonal interaction perception of CCI related positively to perceived value (*b* = 0.42, SE = 0.04, *p* < 0.01, 95% CI [0.39, 0.61]: H1b supported), and that perceived value related positively to purchase intention (*b* = 0.27, SE = 0.05, *p* < 0.01, 95% CI [0.15, 0.38]: H2 supported).

Through mediation analysis, the researchers found that direct effect of CAI on purchase intention (*b* = 0.48, SE = 0.04, *p* < 0.01, 95% CI [0.38, 0.58]) and indirect effect of CAI on purchase intention through perceived value were both significant (*b* = 0.10, SE = 0.03, 95% CI [0.04, 0.16]: H3a supported), indicating their partial mediating effects (Table 3). Similarly, both direct effect of CCI on purchase intention (*b* = 0.50, SE = 0.05, *p* < 0.01, 95% CI [0.39, 0.61]) and indirect effect of CCI on purchase intention through perceived value were significant (*b* = 0.11, SE = 0.03, 95% CI [0.05, 0.19]: H3b supported), also proving their partial mediating effects (Table 4).

**Table 3:** Mediation result (CAI → perceived value → purchase intention).

	**Perceived value (*M*)**				**Purchase intention (*Y*)**			
	**Coeff.**	**SE**	***t*-Value**	**Value of *p***	**Coeff.**	**SE**	***t*-Value**	**Value of *p***
CAI(X1)	0.31	0.04	6.83	<0.01	0.48	0.04	9.65	<0.01
Age	−0.024	0.06	−0.39	0.69	−0.01	0.06	−0.20	0.83
Experience	0.07	0.04	1.57	0.11	−0.05	0.04	−1.02	0.30
Perceived value (*M*)	–	–	–	–	0.32	0.05	9.65	<0.01
Constant	2.60	0.24	10.77	<0.01	0.81	0.28	2.87	<0.01
Model summary	*R*^2^ = 0.12 *F* = 16.40 *p* < 0.01				*R*^2^ = 0.31 *F* = 47.90 *p* < 0.01			

**Table 4:** Mediation result (CCI → perceived value → purchase intention).

	**Perceived value (*M*)**				**Purchase intention (*Y*)**			
	**Coeff.**	**SE**	***t*-Value**	**Value of *p***	**Coeff.**	**SE**	**t-Value**	**Value of *p***
CCI(X2)	0.42	0.04	9.22	<0.01	0.50	0.05	9.17	<0.01
Age	−0.02	0.06	−0.43	0.66	−0.01	0.06	−0.27	0.78
Experience	0.04	0.04	0.96	0.33	−0.08	0.05	−1.70	0.08
Perceived value (*M*)	–	–	–	–	0.27	0.05	4.75	<0.01
Constant	2.24	0.23	9.67	<0.01	0.99	0.28	3.52	<0.01
Model summary	*R*^2^ = 0.19 *F* = 29.29 *p* < 0.01				*R*^2^ = 0.33 *F* = 45.17 *p* < 0.01			

With Hayes’ Process macro (Model 7), the researchers then examined the moderated mediation model by perceived value (Hayes, 2013). Firstly, whether presence moderates the relationship between perceived value and CAI and CCI was investigated into.

**Figure 2:**
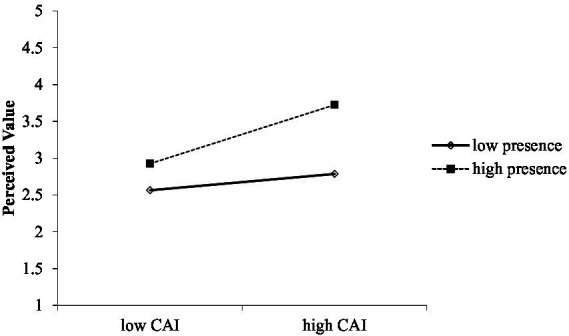
Perceived value based on CAI and presence.

In Table 5, the bootstrap result at a 95% confidence interval indicated that interaction effect of CAI and presence on perceived value was significant (*β* = 0.14, *p* < 0.01). The change in R squared due to the interaction effect was Δ*R*^2^ = 0.02, *p* < 0.01. The simple slopes test also indicated that the relationship between CAI and perceived value was stronger and more significant at +1 standard deviation above the mean value (*β* = 0.12, CI [0.24, 0.53]) than that at −1 standard deviation below the mean value of the moderator presence (*β* = 0.03, CI [0.00, 0.23]). In addition, the moderation graph for Figure 2 demonstrated that strong presence, as opposed to low presence, enhances the relationship between CAI and perceived value. H4a was thereby supported.

**Table 5:** Moderated mediation result (CAI × presence → perceived value → purchase intention).

		***β***	**SE**	Δ***R***^**2**^	**Decision**
H4a	Constant	3.85**	0.04		
	CAI → PV	0.25**	0.05		
	Presence → PV	0.32**	0.06		
	CAI × Presence → PV	0.14**	0.04	0.02	Accepted
Conditional effects of (Presence) at M ± 1 S.D. (slope test)	**Effect**	**SE**	**LL95% CI**	**UL95% CI**	
Presence Low −1 SD (−0.94)	0.03	0.02	0.00	0.23	
Presence Medium M (0.00)	0.08	0.02	0.15	0.35	
Presence High +1 SD (0.94)	0.12	0.04	0.24	0.53	

*N* = 359; **p < 0.01. CAI, consumer-anchor interaction; PV, perceived value.

**Figure 3:**
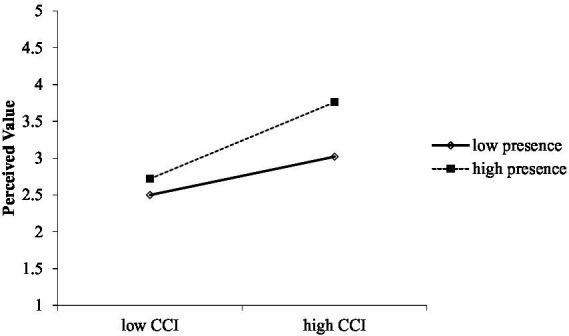
Perceived value based on CCI and presence.

As shown in Table 6, the bootstrap result at a 95% confidence interval demonstrated that interaction effect of CCI and presence on perceived value was significant (*β* = 0.13, *p* < 0.01). The change in *R* squared due to the interaction effect was Δ*R*^2^ = 0.02, *p* < 0.01. The simple slopes test also indicated that the relationship between CCI and perceived value was stronger and more significant at +1 standard deviation above the mean value (*β* = 0.52, CI [0.37, 0.67]) than that at −1 standard deviation below the mean value of the moderator presence (*β* = 0.26, CI [0.15, 0.37]). Besides, the moderation graph for Figure 3 showed that strong presence, as opposed to low presence, enhances the relationship between CCI and perceived value. Hence, H4b was proved tenable.

**Table 6:** Moderated mediation result (CCI × Presence → perceived value → purchase intention).

		*β*	SE	Δ*R*^2^	Decision
H4b	Constant	3.85**	0.04		
	CCI → PV	0.39**	0.05		
	Presence → PV	0.24**	0.05		
	CCI × Presence → PV	0.13**	0.04	0.02	Accepted
Conditional effects of (Presence) at M ± 1 S.D. (slope test)	**Effect**	**SE**	**LL95% CI**	**UL95% CI**	
Presence Low −1 SD (−0.94)	0.26	0.05	0.15	0.37	
Presence Medium M (0.00)	0.39	0.05	0.28	0.49	
Presence High +1 SD (0.94)	0.52	0.07	0.37	0.67	

*N* = 359; **p < 0.01. CCI, consumer-consumer interaction; PV, perceived value.

## 4. Discussion

The study has come up with 4 research questions at the beginning and investigated into them by analyzing the research data. The conclusions are as follows.

For the first research question, the results of the study demonstrate that CAI and CCI have a positive effect on consumers’ perceived value (H1a and H1b). Li et al. also concluded similar research results, and illustrated that consumers acquire different perceived values when interacting with others online (Li et al., 2021). It is also found that there is a substantial influence of interpersonal interaction on consumer perceived value. However, there exist some researches showing conflicting results. Liu and Congcong (2018) discovered that self-oriented interaction has a significant negative impact on perceived value while emotional-oriented interaction and task-oriented interaction have a considerable positive influence. The contradiction may have arisen from the fact that this study examined the consumers’ interpersonal interaction rather than perceived interpersonal interaction. Another reason might be that situational factors (Holiday Tour vs. E-commerce Live Broadcasting) could lead to different final results. In this regard, it is necessary for future researches to verify the relationship between consumers’ interaction perception and perceived value.

The research results can also prove the second hypothesis since it is found that purchase intention is affected by consumers’ perceived value (H2). Consumers’ purchase intention refers to the comprehensive purchase intention from consumers after their taking into account the anticipated family income, expected price, and anticipated revenue (the income from purchasing products; Ponte et al., 2015). The study proves a clear relationship between consumers’ perceived value and purchase intention, considering their close conceptual relationship.

For the third hypothesis, this study verified the mediating role of perceived value on interpersonal interaction and purchase intention (H3a and H3b). Based on the live broadcasting of brand shops, a similar research of Wu and Guan (2021) revealed that perceived value and customers’ engagement both play the role of a mediator.

Information systems are frequently used by e-commerce organizations to encourage interactive communication between sellers and buyers (Kim et al., 2021). Consumers’ purchase intention increases with the relevance and richness of the interpersonal interaction as well as their volume. E-commerce platforms are more interactive than social media. When it comes to consumers’ interaction, interpersonal interactions are quite important. As a result, improving interpersonal interaction might be helpful for business promotion in an industry that uses live e-commerce broadcasting.

For the final hypothesis, we tested the moderating effect of presence on the relationship between interpersonal interaction and perceived value (H4a and H4b). The significance of interpersonal interaction was thus proved. As interpersonal interaction is frequent in both CAI and CCI, the perceived value of the consumers is significantly higher since anchors will communicate with the audience based on their bullet screen and virtual gifts. In this regard, streaming media offer a interaction function for users, thereby evoking their strong sense of participation and a positive perceived value (Lin and Sun, 2014).

Above all, all the hypotheses are tenable in this way. It is concluded that both of CAI and CCI have a significant effect on purchase intention by influencing consumers’ perceived value. Meanwhile, presence functions as a moderator in perceived value and interpersonal interaction. Presence is not only a technical problem, but also a management problem since it is an essential factor to maintain competitive advantages in the industry. The results of the study indicate that it is conducive for e-commerce enterprises to improve consumers’ interpersonal interaction and presence when developing live broadcasting shopping services. In the process, e-commerce enterprises are expected to attach great importance to interactive features and impressive communication experience in order to promote their purchasing behaviors.

## 5. Conclusion

This paper contributes to the existing literature in interpersonal interaction of e-commerce live broadcasting by confirming a mediating process and identifying a moderating process in the links among interpersonal interaction perception, perceived value, and purchase intention. The findings indicate the critical role of perceived value in the association between interpersonal interaction perception and purchase intention. Although some researches identified a relationship between interpersonal interaction perception and consumers’ purchase intention, the mediating role of consumer perceived value is still worth investigating in relation to purchase intention. Moreover, the presence will function as a critical moderating factor to explain the link between interpersonal interaction perception and perceived value. Presence has been investigated in relation to viewers’ watching live streaming (Chen and Liao, 2022); however, no study could identify the moderating effect of presence in the context of interpersonal interaction and perceived value. In addition, the results of this study enhance the understanding of interpersonal interaction based on the S-O-R model and flow theory.

Regarding practical implications, enterprises need to recognize that in e-commerce live broadcasting, consumers can form purchase intention through consumer perceived value. Previous studies indicate that by enhancing consumers’ perceived value, organizations can achieve a competitive advantage and guarantee their sustainability (Hervás et al., 2020). The study shows that enhanced perceived value enables a stronger purchase intention, which will eventually raise customers’ loyalty (Kim et al., 2021). From the perspectives of consumers and their enterprises, perceived value can increase the satisfaction of the users and shape the brand image, respectively (Li and S, 2022).

Additionally, enterprises in the e-commerce live broadcasting industry need to enhance interpersonal interaction perception among consumers, because it brings better vital and loyal consumers. Under e-commerce live broadcasting mode, consumers’ speeches and purchase behaviors will be displayed in real time in the live broadcasting room, which may be observed and evaluated by others at any time. Hence, the increase of presence possibly triggers a stronger fear of evaluation among consumers, thus promoting online consumption. The role of interpersonal interaction perception can benefit beyond enterprises and customers. Customers will receive more accurate and high-quality service information by interacting with interactive and motivated anchors (Li and S, 2022). Hence, enterprises that can retain these anchors may have a competitive advantage in human resources within the e-commerce live broadcasting industry (Xie et al., 2019). Furthermore, such competitive advantages can guide enterprises and anchors to improve the quality of the live broadcasting, thus achieving their marketing targets (Werner and Balkin, 2021).

This study contributes to filling in the gap in the existing researches on interpersonal interaction, and also provides practical implications to enterprises in the live broadcasting industry. However, there are several limitations. Firstly, the paper only explored one of features of live broadcasting – the influence of interpersonal interaction. E-commerce live broadcasting is an increasingly mature product of the live broadcasting technology, which integrates various features of new technology. Hence, future studies may investigate into e-commerce live broadcasting combining other features. Secondly, the scope of the participants is limited for the study only involved Chinese e-commerce live broadcasting consumers. To achieve generalizability, the scope of future studies can be extended to other industries in different countries. Thirdly, we only used interpersonal interaction as an independent variable, omitting other potential variables. Other variables such as human-computer interaction also have the certain effect on purchase intention. Finally, consumer perceived value can be divided into different types of value – functional value, hedonic value, and social value (Soutar, 2001), but this study employed overall perceived value singularly as a mediator. Further studies will test whether different types of perceived value can improve the connection between interpersonal interaction and purchase intention.

## Data availability statement

The original contributions presented in the study are included in the article/supplementary material, further inquiries can be directed to the corresponding author.

## Author contributions

XM, JJ, and YL contributed to conception and design of the study, wrote the first draft of the manuscript, and contribute to the data collection and analysis. All authors contributed to the article and approved the submitted version.

## Funding

This work is supported by Graduate School of Konkuk University.

## Conflict of interest

The authors declare that the research was conducted in the absence of any commercial or financial relationships that could be construed as a potential conflict of interest.

## Publisher’s note

All claims expressed in this article are solely those of the authors and do not necessarily represent those of their affiliated organizations, or those of the publisher, the editors and the reviewers. Any product that may be evaluated in this article, or claim that may be made by its manufacturer, is not guaranteed or endorsed by the publisher.

## Footnotes

^1^ http://www.sojump.com
